# Supplementary material for: Coriolic Acid (13-(S)-Hydroxy-9Z, 11E-octadecadienoic Acid) from Glasswort (Salicornia herbacea L.) Suppresses Breast Cancer Stem Cell through the Regulation of c-Myc
Source: Molecules. 2020 Oct 26;25(21):4950. doi: 10.3390/molecules25214950 (PMC7663198; doi:10.3390/molecules25214950)
Supplement: Supplementary file 1 [file molecules-25-04950-s001.pdf]

Table S1. Specific Real-time RT-qPCR primer sequences containing *Nanog, CD44, Oct4*, c-myc, and *β-actin* genes

Genes	Primers
Nanog	Forward: 5′-ATGCCTCACACGGAGACTGT-3′<br>Reverse: 5′-AAGTGGGTTGTTTGCCTTTG-3′
CD44	Forward: 5′-AGAAGGTGTGGGCAGAAGAA-3′<br>Reverse: 5′-AAATGCACCATTTCCTGAGA-3′
Oct4	Forward: 5′-AGCAAAACCCGGAGGAGT-3′<br>Reverse: 5′-CCACATCGGCCTGTGTATATC-3′
c-myc	Forward: 5′-AATGAAAAGGCCCCCAAGGTAGTTATCC-3′<br>Reverse: 5′-AGCAAAACCCGGAGGAGT-3′
β-actin	Forward: 5’-TGTTACCAACTGGGACGACA-3’<br>Reverse: 5’-GGGGTGTTGAAGGTCTCAAA-3

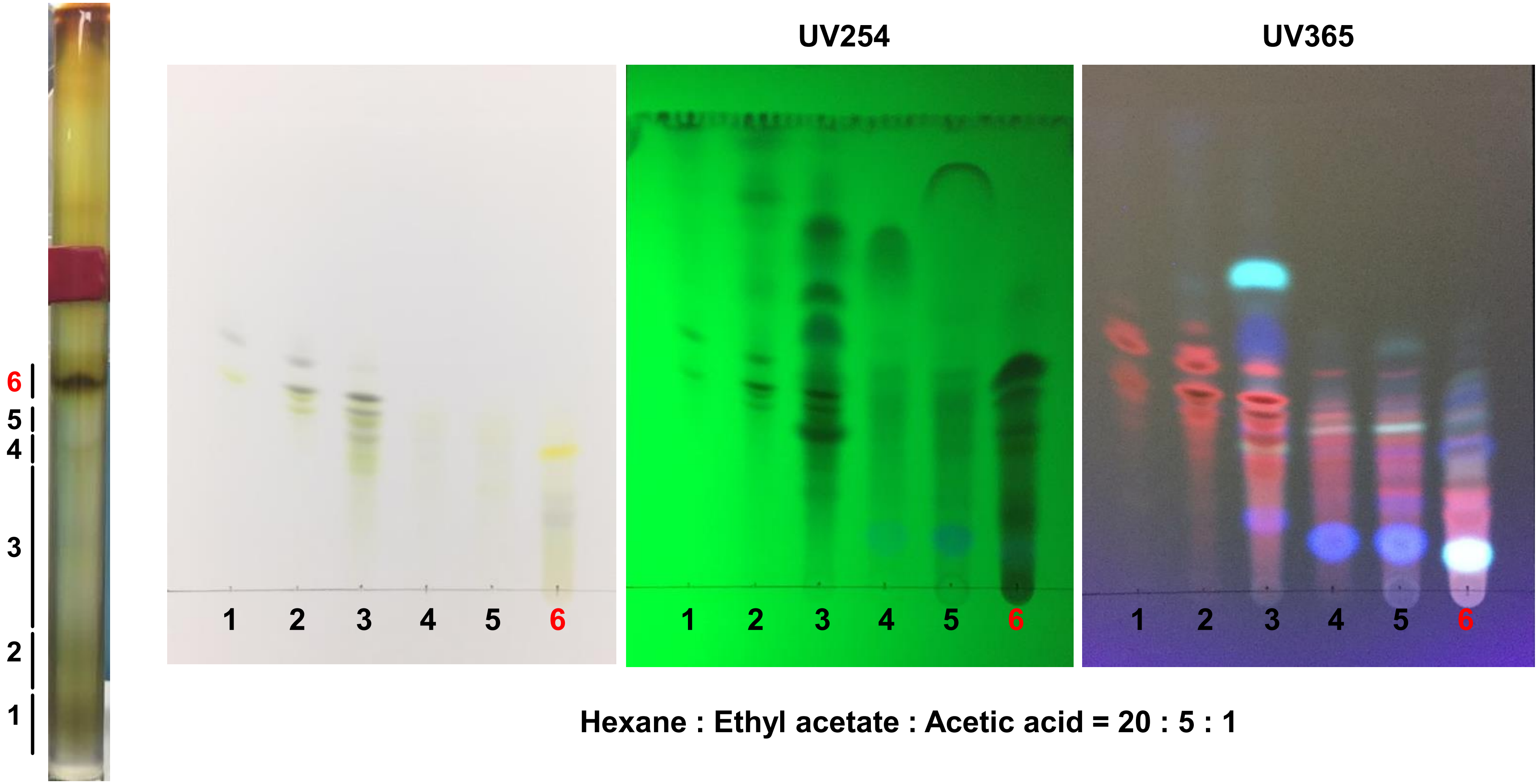

Figure S1. Purification procedure of the inhibitor of mammosphere formation derived from glasswort extracts using SiO_2 gel chromatography eluted with $CHCl_3$: MeOH (30:1).

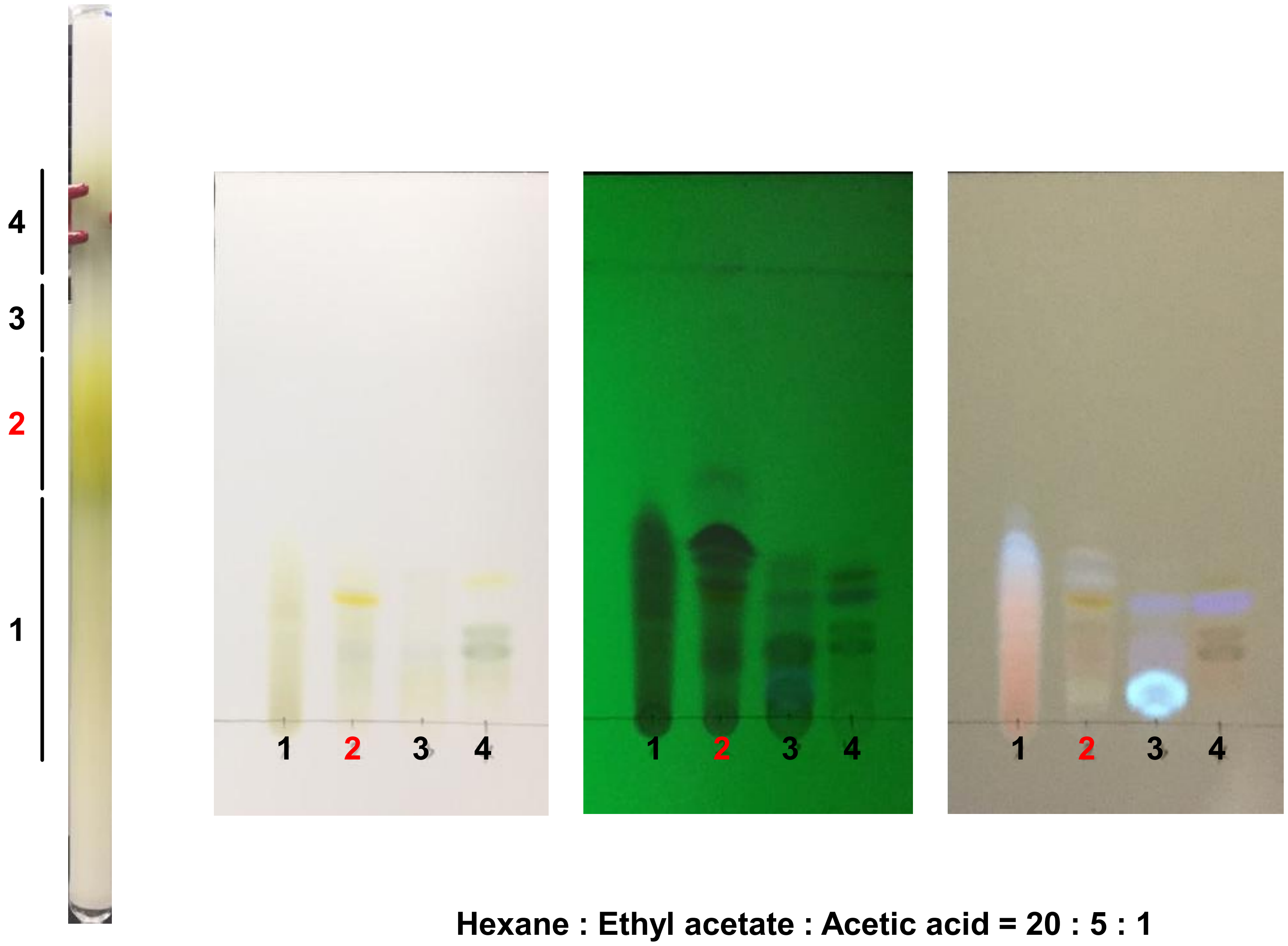

Figure S2. Purification procedure of the inhibitor of mammosphere formation derived from glasswort extracts using sephadex LH-20 gel chromatography eluted with MeOH.

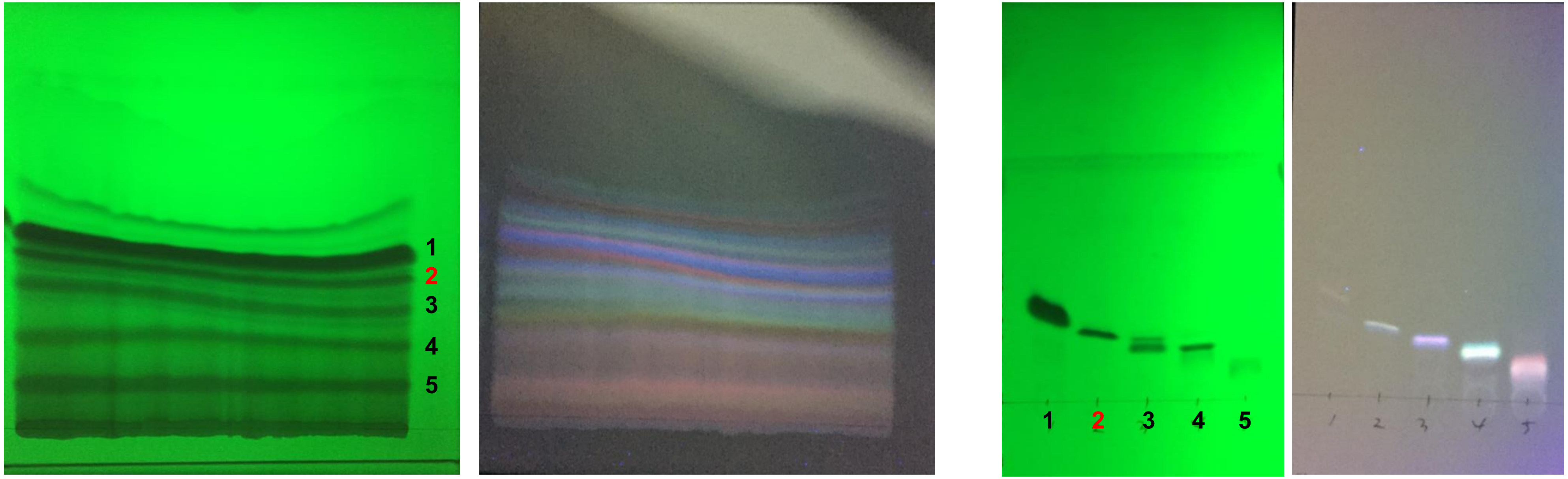

Hexane : Ethyl acetate : Acetic acid = 15 : 5 : 1

Hexane : Ethyl acetate : Acetic acid = 20 : 5 : 1

Figure S3. Purification procedure of the inhibitor of mammosphere formation from glasswort extracts using preparative thin layer chromatography with Hexane:Ethyl acetate:Acetic acid (15:5:1).

HPLC

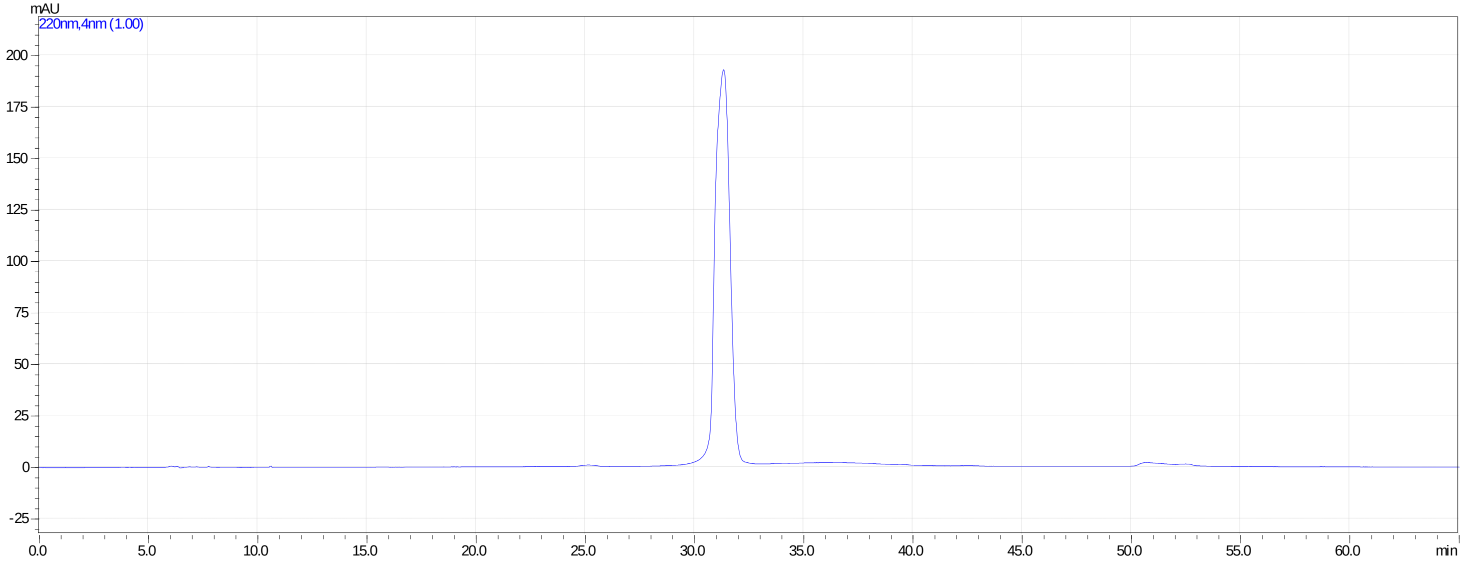

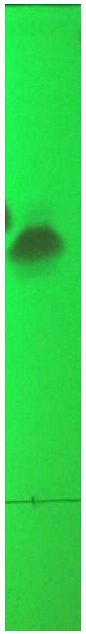

Figure S4. High performance liquid chromatography of purified sample

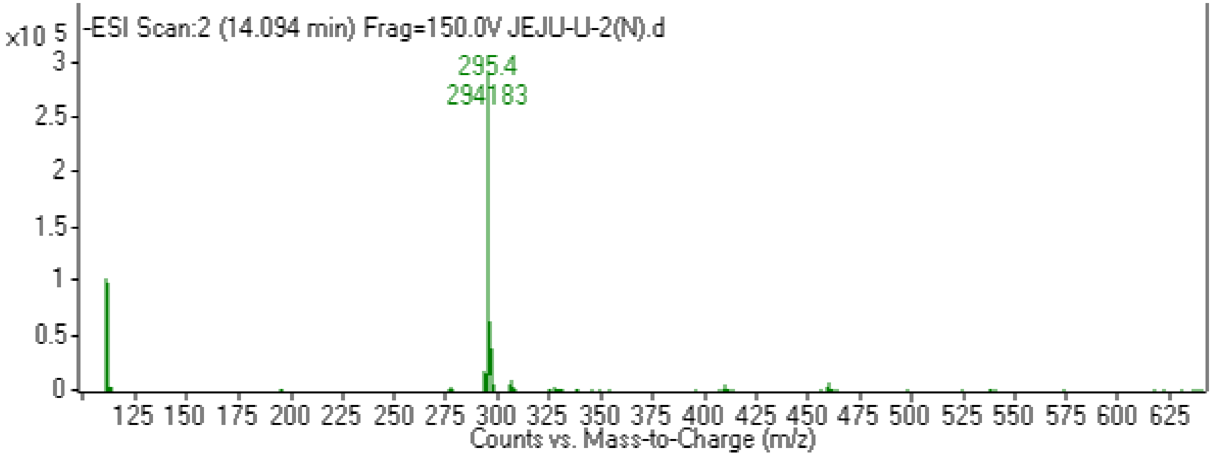

Figure S5. ESI mass spectrometry of the purified sample

H-NMR

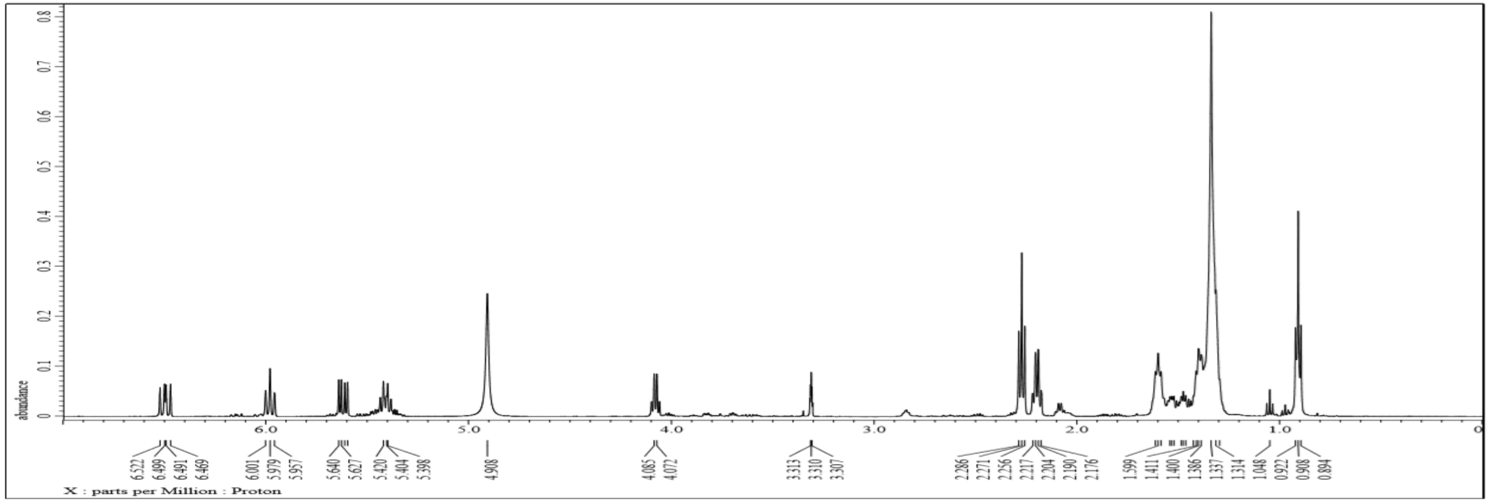

C-NMR

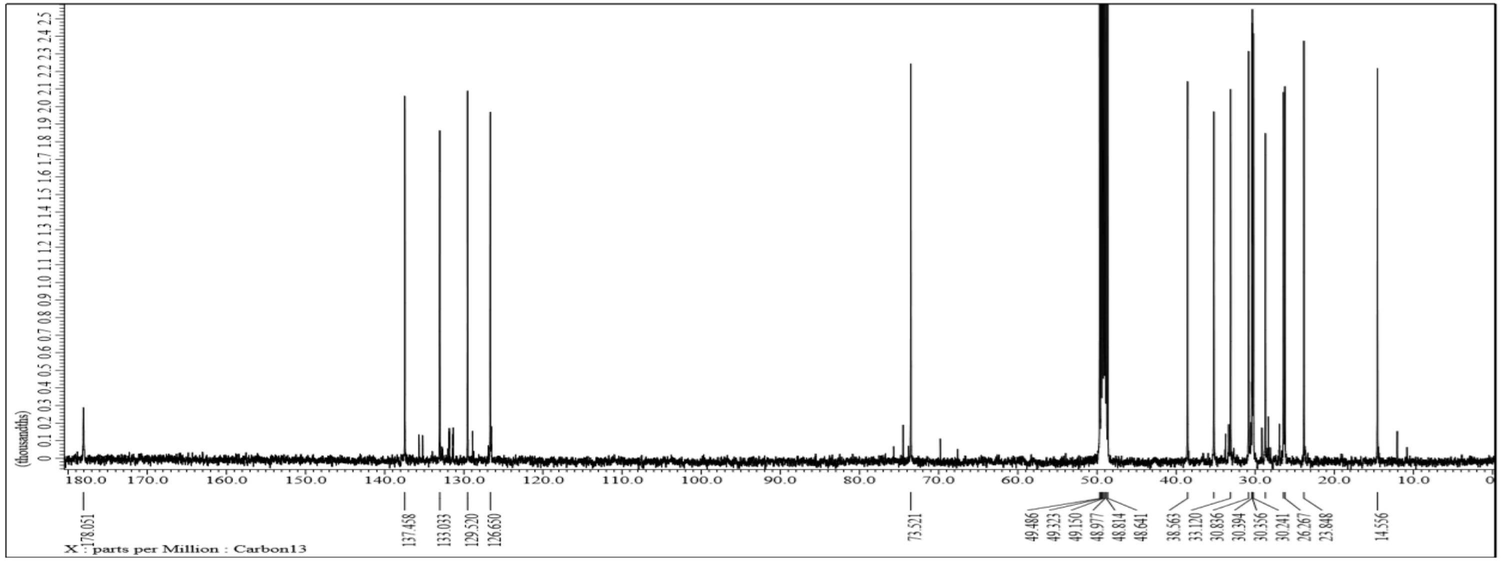

Figure S6. ^{1}H NMR and ^{13}C NMR spectra of the purified sample

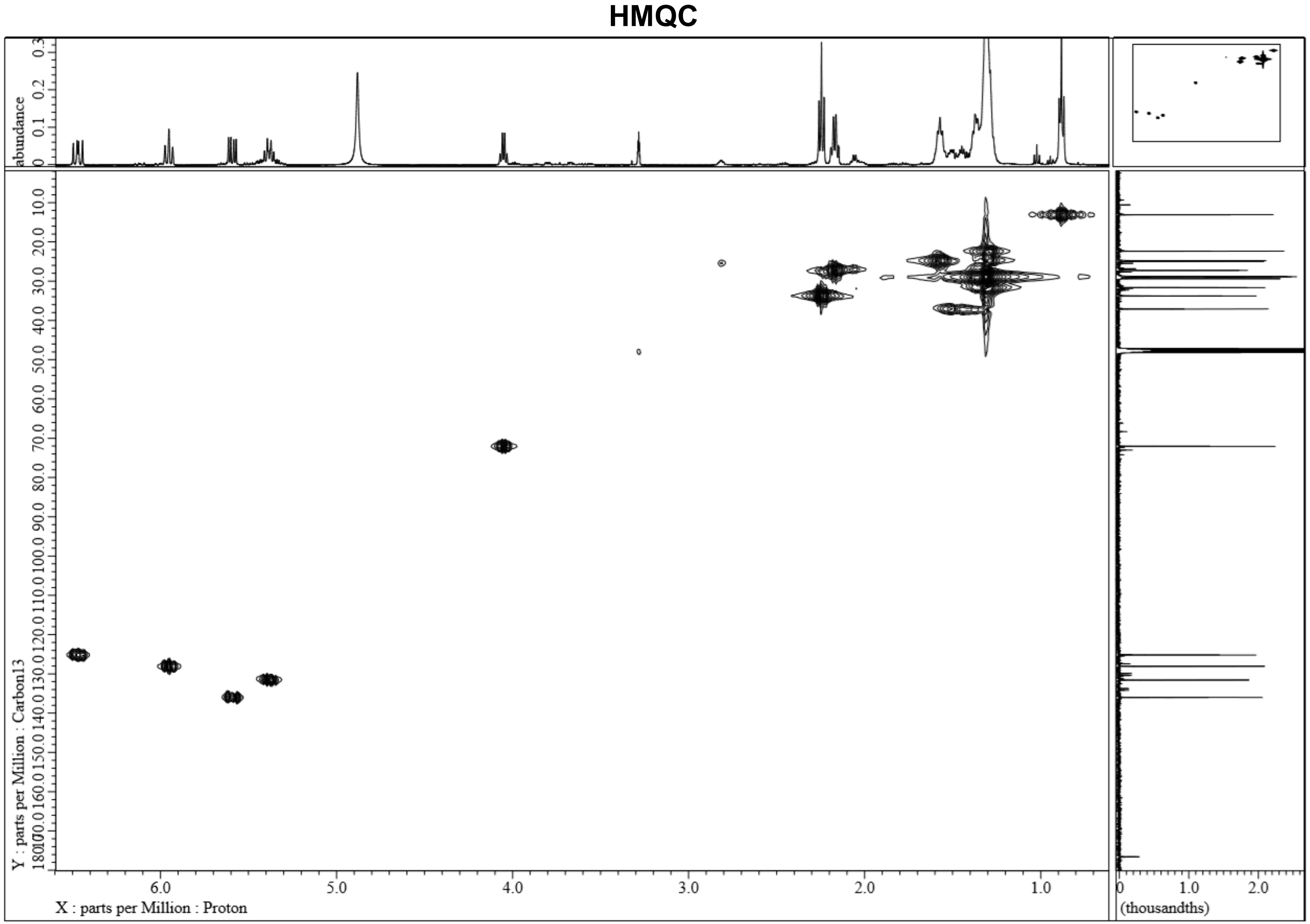

Figure S7. HMQC spectrum of the purified sample

COSY

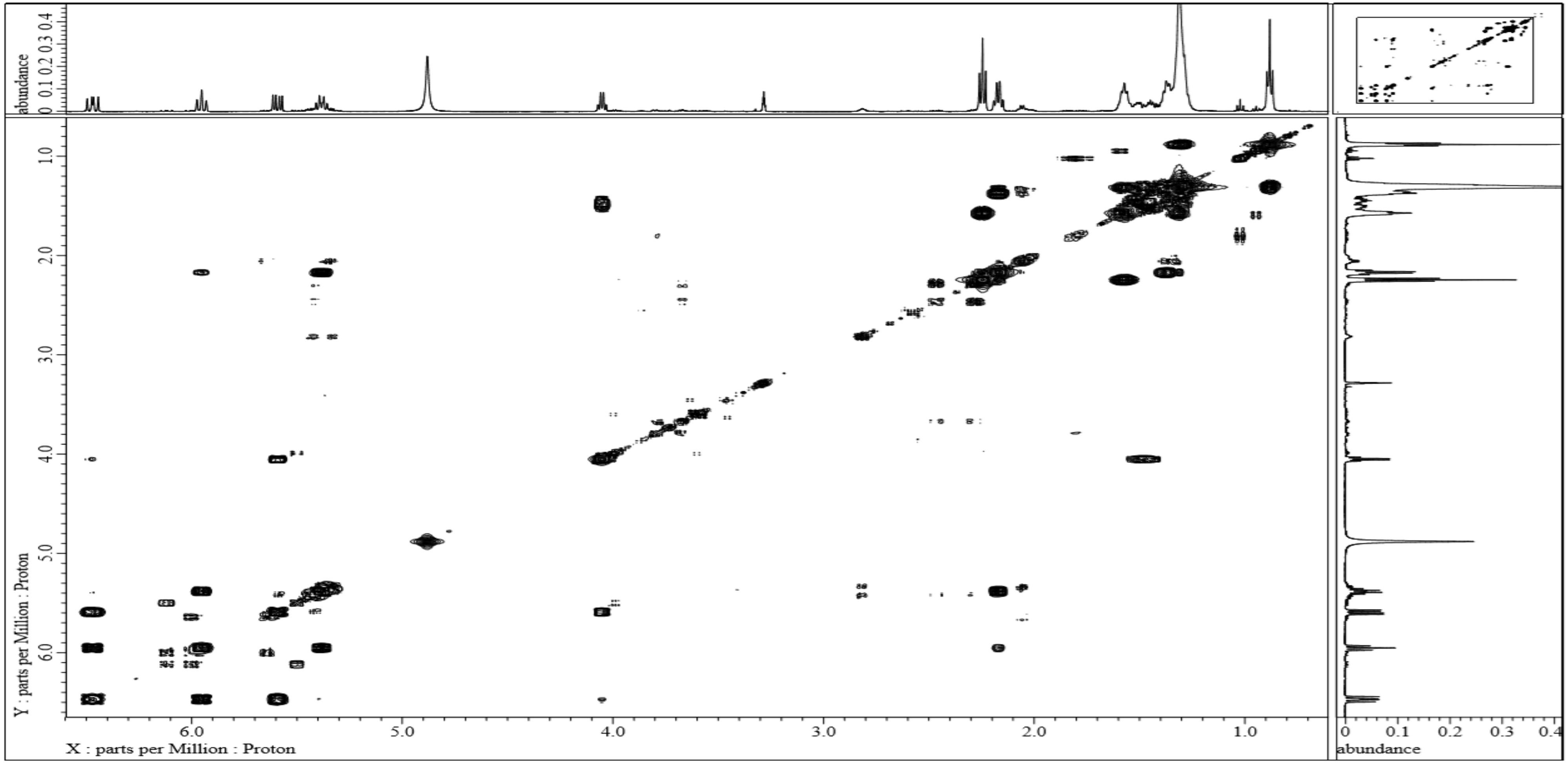

Figure S8. ^1H-^1H COSY spectrum of the purified sample

HMBC

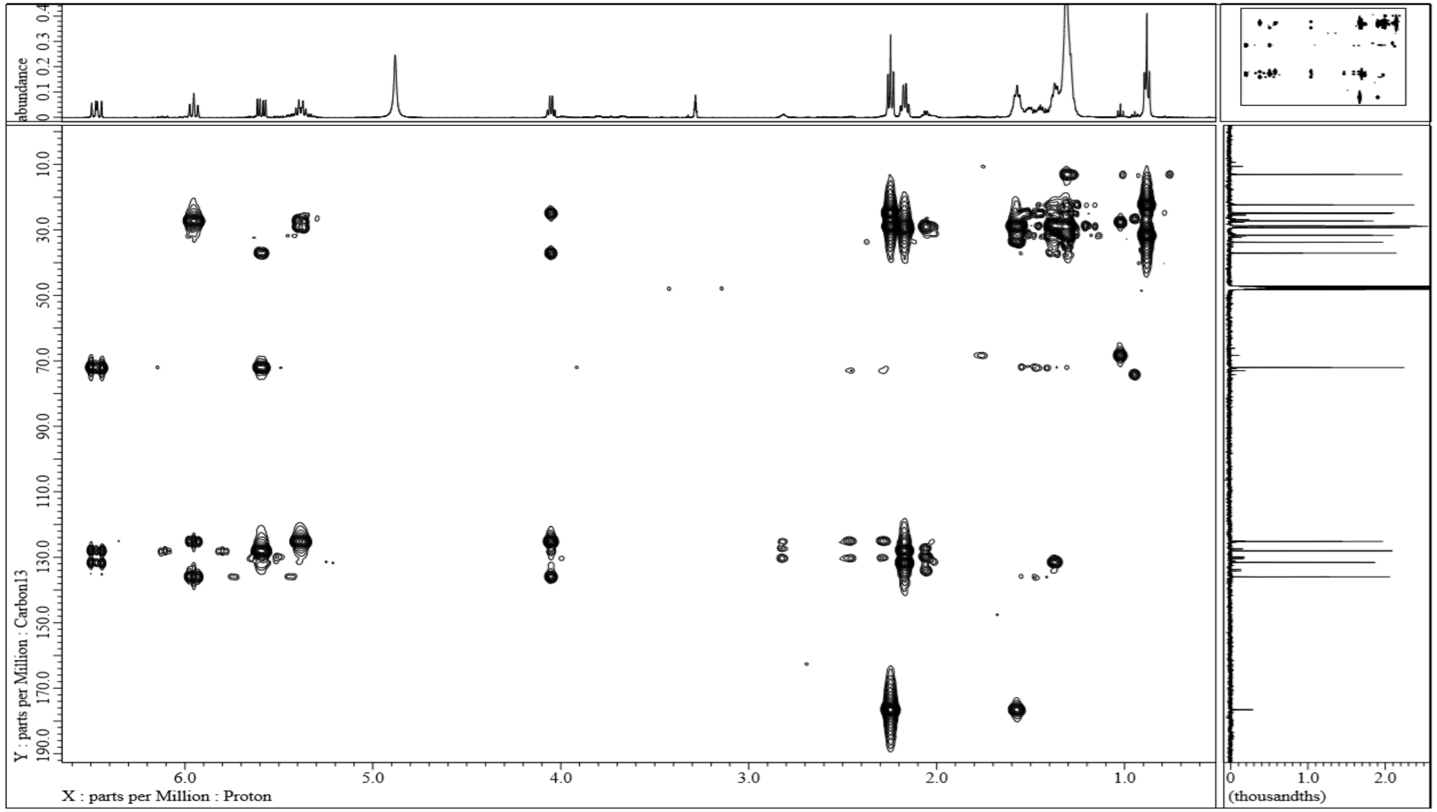

Figure S9. HMBC spectrum of the purified sample

A

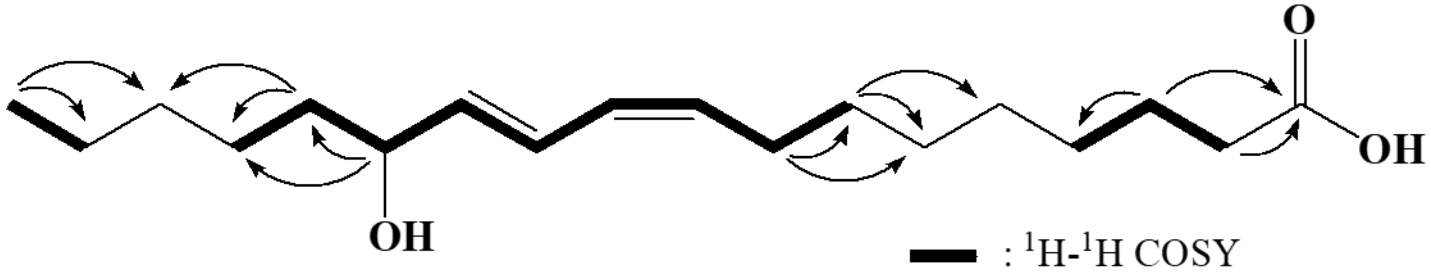

B

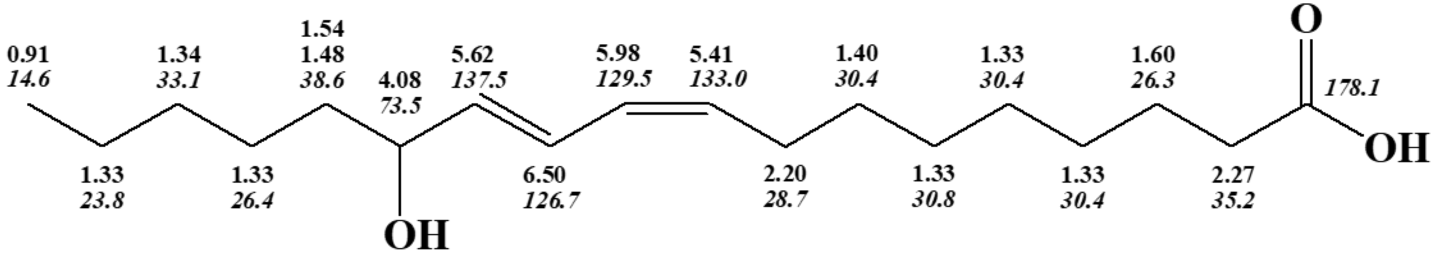

Coriolic acid (= 13-HODE, 13-Hydroxylinoleic acid, α-Artemisolic acid, (9Z,11E)-13-Hydroxy-9,11-octadecadienoic acid)
Molecular formula: C18H32O3, Molecular weight: 296 (m/z 295 [M-H]-)

Figure S10. Two-dimensional NMR data (A) and ^{1}H and ^{13}C (italics) peaks assignments (B) of the purified sample
